# Novel Magnetic Covalent Organic Frameworks Fabricated Through In Situ Synthesis and Assembly for the Efficient Extraction and Enrichment of Six Amide Herbicides

**DOI:** 10.3390/molecules31111940

**Published:** 2026-06-03

**Authors:** Haiyue Sun, Yihan Luo, Jingyu Zhao, Jiaying Liu, Jingli Yu, Junhong Xin

**Affiliations:** 1 Innovative Institute of Chinese Medicine and Pharmacy, Shandong University of Traditional Chinese Medicine, Jinan 250355, China; 18353453658@163.com (H.S.); 15139859625@163.com (J.Z.); 2 College of Health Sciences, Shandong University of Traditional Chinese Medicine, Jinan 250355, China; 18870755658@163.com (Y.L.); d40538351@163.com (J.L.); 13310627802@163.com (J.Y.); 3 Key Laboratory of Traditional Chinese Medicine Classical Theory, Ministry of Education, Shandong University of Traditional Chinese Medicine, Jinan 250355, China

**Keywords:** magnetic covalent organic frameworks, magnetic solid-phase extraction, gas chromatography-tandem mass spectrometry, amide herbicides, adsorption mechanism

## Abstract

Magnetic covalent organic frameworks (MCOFs) offer efficient adsorption via designable pore channels and active sites, along with rapid magnetic separation due to their intrinsic superparamagnetism. However, physical mixing or non-covalent assembly often leads to weak binding, causing the leaching or detachment of magnetic components during use, and compromises the well-defined crystallinity of the COF. In this study, we employed an in situ synthesis strategy at room temperature based on amidation and Schiff base reactions to fabricate a magnetic TAPT-DHTA-COF with good crystallinity and superparamagnetism. This material was used as a magnetic solid-phase extraction (MSPE) adsorbent to establish an MSPE-GC-MS/MS method for the determination of amide herbicides (AHs). The TAPT-DHTA-COF is rich in hydroxyl groups, which form strong hydrogen bonds with the polar AH molecules. In a green tea matrix, six AHs showed good linearity within the concentration range of 1–500 ng g^−1^, with correlation coefficients ranging from 0.9910 to 0.9982. The limits of detection were between 0.25 and 0.73 ng g^−1^, spiked recoveries ranged from 80.1% to 94.8%, and relative standard deviations were below 6.2%. This work offers an improved synthesis strategy for novel magnetic COFs and insights into their application in adsorbing polar pesticides.

## 1. Introduction

As a beverage cherished worldwide for its distinctive aroma and health benefits, tea requires strict pesticide residue testing during production to ensure its quality and safety [1,2]. Among these, AHs constitute a class of highly effective, highly selective contact pesticides characterized by broad-spectrum efficacy and ease of application [3]. However, excessive use of such pesticides tends to leave residues in surface water and soil, subsequently posing risks to human health through the food chain [4]. AHs exhibit certain genotoxicity. For instance, butachlor can induce cancer in animals, chromosomal aberrations in human lymphocytes, and immunotoxic effects [5]. Acetochlor has been found to possess thyroid-disrupting properties [6] and has been classified by the United States Environmental Protection Agency as a Group B-2 carcinogen. Most AHs exhibit moderate volatility and a degree of thermal stability; hence, they are typically analyzed using GC or GC-MS [7]. Owing to the complex nature of tea leaf matrices, which are rich in polyphenols, alkaloids, pigments, and polysaccharides [8], these substances readily undergo co-extraction and matrix effects during analysis, such as polyphenolic compounds [9] causing signal suppression or enhancement, which significantly compromises the accuracy and sensitivity of detection. Therefore, establishing an effective sample preparation method is a critical step in the entire analytical process, being of paramount importance for enhancing detection specificity, sensitivity, and efficiency [10].

Conventional pretreatment techniques are typically characterized by cumbersome operations, time-consuming procedures, and the substantial consumption of organic solvents [11,12]. MSPE technology enables the rapid separation of adsorbents through the application of an external magnet, offering advantages such as high extraction efficiency, short operation times, reduced solvent consumption, and minimal adsorbent usage [13,14,15]. Magnetic adsorbents are pivotal in determining the efficiency of MSPE [16]; hence, the selection of highly effective magnetic adsorbents is of paramount importance. In recent years, numerous materials of MSPE have been developed and utilized [17,18]. Multi-walled carbon nanotubes are porous, graphite-based cylindrical nanomaterials [19] characterized by a large specific surface area and high chemical stability [20,21]. Ma et al. [22] employed multi-walled carbon nanotubes as adsorbents to successfully enrich polycyclic aromatic hydrocarbons, pyrazole, and pyrrole pesticides in environmental water samples. However, research has revealed that its pore structure is disordered and uncontrollable, with the adsorption of specific target substances primarily relying on non-specific π-π interactions and hydrogen bonding. This mechanism may prove insufficiently selective when confronted with more complex sample matrices, readily co-adsorbing other interfering substances. Magnetic metal–organic framework materials, owing to their outstanding tunable pore structure [23], demonstrate considerable application potential in the field of sample pretreatment [24]. Li et al. [25] synthesized a Fe_3_O_4_ core–shell nanocomposite coated with a bimetallic (Mg/Zn) MOF-74 framework for the rapid, highly sensitive quantitative detection of aflatoxin B_1_ in grain samples. However, the synthesis of this material requires relatively high temperatures, and in complex sample matrices, it may exhibit insufficient water stability and structural collapse. MCOFs demonstrate potential owing to their high stability, designable functionality, and outstanding adsorption properties [26,27,28]. As demonstrated by Guo et al. [29], they achieved the efficient, stable, and reproducible enrichment and detection of trace benzene urea herbicides in plant beverages. This was accomplished by designing a magnetic carboxyl quinoline covalent organic framework material (Fe_3_O_4_@CQ-COF) based on an imine cyclisation reaction and coupling it with MSPE combined with HPLC detection technology. Jiang et al. [30] designed a COF-functionalized magnetic MXene nanocomposite (CoFe_2_O_4_@Ti_3_C_2_@TAPB-TFTA) based on Schiff base reactions, achieving the efficient enrichment and sensitive detection of ten trace organophosphorus and organochlorine pesticides in tea samples. This was accomplished using MSPE coupled with GC-MS/MS detection. However, the following issues may arise during the preparation of MCOF materials. First, achieving stable composite formation between magnetic nanoparticles (MNPs) and COFs remains a core challenge, as MNPs bound by physical adsorption readily detach from the framework, leading to the loss of components. Second, the synthesis of many classic COFs requires elevated temperatures (typically 120–200 °C) to drive reversible reactions and form ordered crystalline structures. This process often necessitates pressure-resistant vessels and strict anhydrous, oxygen-free conditions, thereby increasing process complexity and energy consumption. Such requirements are detrimental to the environmentally sustainable, large-scale production of these materials. Third, COFs predominantly constructed from aromatic monomers are inherently hydrophobic, resulting in the inadequate dispersion of magnetic materials in aqueous solutions. This renders numerous internal active sites inaccessible, thereby compromising mass transfer efficiency.

To address the aforementioned issues, this study optimized the reaction conditions to prepare uniformly shaped carboxylated magnetic spheres that remain stably dispersed in aqueous solutions. Magnetic TAPT-DHTA-COF was synthesized at room temperature by first covalently grafting the monomer 2,4,6-tris(4-aminophenyl)-1,3,5-triazine (TAPT) onto magnetic spheres via an amide reaction, followed by its polycondensation with the second monomer 2,5-dihydroxyterephthalaldehyde (DHTA) through a Schiff base reaction. The covalent anchoring strategy via amide bonds effectively suppressed magnetic bead self-aggregation, enabling the uniform and stable loading of MNPs within the COF matrix. By introducing carboxylated magnetic spheres and utilizing the polar functional groups inherent to the COF, the hydrophilicity and aqueous dispersibility of the material were synergistically enhanced, thereby improving its adsorption capacity for amide-type herbicides. Based on this novel MCOF material, a new combined detection method for the qualitative and quantitative analysis of six AHs in tea was established by optimizing magnetic solid-phase extraction conditions and integrating GC-MS/MS technology. The adsorption mechanism between the MCOF material and AHs was also investigated. This research establishes a robust analytical foundation for the compositional and contaminant profiling of tea, thereby supporting the refinement of industry-wide specifications.

## 2. Results and Discussion

### 2.1. Magnetic Sphere Synthesis Optimization

**Figure 1:**
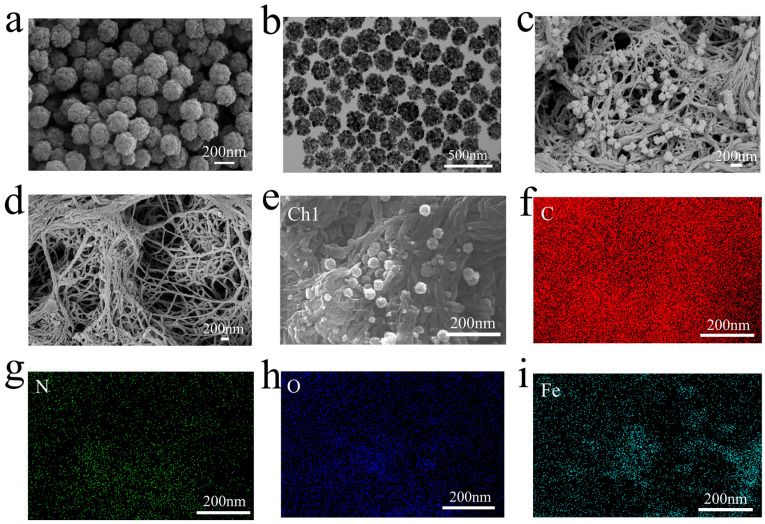
(**a**) SEM and (**b**) TEM images of magnetic sphere synthesized in the aqueous phase. SEM images: (**c**) magnetic TAPT-DHTA-COF 1, (**d**) TAPT-DHTA-COF, and (**e**–**i**) EDS element mappings of magnetic TAPT-DHTA-COF 1.

In the present study, two types of carboxylated magnetic spheres were synthesized. SEM was employed to characterize their morphological features, as presented in Figure 1a, which depicts the product synthesized in the aqueous phase, while Figure S2 corresponds to the material synthesized in the organic phase (ethylene glycol). From the micrographs, distinct differences in surface appearance, morphology, and particle diameter are evident between the two preparations. The magnetic spheres shown in Figure S2 exhibit a larger average diameter compared to those in Figure 1a. Furthermore, the particles synthesized in the aqueous phase present a more uniform and regular morphology, with minimal fragmentation observed.

The water dispersibility of both magnetic sphere types was evaluated using the following procedure. Each material (10 mg) was weighed into separate glass tubes, followed by the addition of 20 mL of distilled water. The mixtures were subjected to ultrasonic dispersion for 10 min and subsequently allowed to stand at room temperature. Tube 1 contained the aqueous-phase-synthesized magnetic spheres, while tube 2 contained the organic-phase-synthesized counterparts. As illustrated in Figure S3, immediately after sonication, both types of particles were uniformly dispersed in the aqueous medium. However, upon prolonged standing, marked differences in the colloidal stability became apparent. The dispersion in tube 2 exhibited obvious sedimentation and phase separation after 4 h, with the stratification becoming more pronounced following 24 h of standing. In contrast, the magnetic spheres in tube 1 maintained stable dispersion in the aqueous phase with no observable sedimentation or phase separation even after 24 h.

In comparison to the ethylene glycol-based organic solvent synthesis system, the aqueous-phase method offers greater economic efficiency and environmental sustainability. In addition, the magnetic spheres synthesized via the aqueous-phase method exhibit a more uniform morphology. As shown in the TEM image (Figure 1b), the diameters of these magnetic spheres predominantly fall within the range of 200–250 nm. Dispersibility experiments further demonstrate that aqueous-phase-synthesized magnetic spheres possess enhanced water dispersibility and maintain stable dispersion in aqueous media. Consequently, the aqueous-phase synthesis method was selected for the preparation of carboxylated magnetic spheres in this study.

### 2.2. Characterization of Magnetic TAPT-DHTA-COF

TAPT-DHTA-COF, magnetic TAPT-DHTA-COF 1, and magnetic TAPT-DHTA-COF 2 were synthesized at room temperature. Magnetic TAPT-DHTA-COF 1 was fabricated through an initial amidation step, which covalently linked carboxylated magnetic spheres to the monomer TAPT, followed by a Schiff base reaction between TAPT and DHTA. In contrast, magnetic TAPT-DHTA-COF 2 was solely synthesized via the Schiff base reaction without the preceding amidation step. As shown in Figure S1, the recoveries of the six amide herbicides across the three batches ranged from 87.2% to 102.09%, with inter-batch RSD values ranging from 0.24% to 4.18% (e.g., acetochlor: 0.24%, alachlor: 4.18%, etc.). These low RSD values demonstrate that the synthesis of the MCOF material is highly reproducible, and the method reliability is consistent across different batches. The morphology of the synthesized materials was characterized by SEM. As shown in Figure 1d, TAPT-DHTA-COF exhibits a tubular and interwoven structural morphology. Two distinct magnetic COF composites were obtained by varying the magnetic sphere incorporation methods. Figure 1c presents the SEM image of magnetic TAPT-DHTA-COF 1, while Figure S4 corresponds to magnetic TAPT-DHTA-COF 2. The images reveal that the magnetic spheres in magnetic TAPT-DHTA-COF 2 are densely and irregularly aggregated, and the tubular architecture formed by the covalent linkage of TAPT and DHTA monomers is disrupted during magnetic sphere loading. In comparison, magnetic TAPT-DHTA-COF 1 displays a more uniform dispersion of magnetic spheres, while the underlying tubular structure of the covalent organic framework remains clearly visible, with tube diameters predominantly ranging from 30 to 70 nm. These results indicate that the incorporation of an amidation step promotes a more dispersed attachment of carboxylated magnetic spheres onto the COF framework, thereby reducing magnetic sphere self-aggregation. Consequently, the synthesis route employed for magnetic TAPT-DHTA-COF 1 was selected to prepare the magnetic COF material, which was subsequently utilized as the adsorbent in MSPE. EDS analysis confirmed the homogeneous distribution of C, N, O, and Fe (Figure 1e–i; see also Figure S5 in the Supplementary Materials). These results demonstrate the successful synthesis of the magnetic TAPT-DHTA-COF and indicate its potential suitability as an MSPE adsorbent for sample preparation.

**Figure 2:**
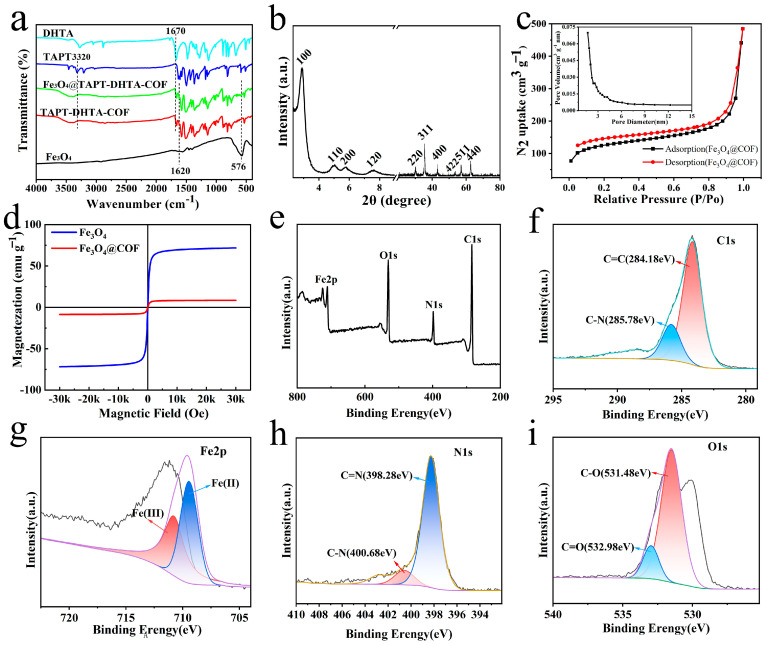
The characterization of magnetic TAPT-DHTA-COF: (**a**) FTIR spectra, (**b**) XRD, (**c**) nitrogen adsorption–desorption isotherms with pore size distributions, (**d**) the magnetization hysteresis curves, (**e**) XPS spectra of magnetic TAPT-DHTA-COF, (**f**) C 1s, (**g**) Fe 2p, (**h**) N 1s, and (**i**) O 1s.

FTIR spectroscopy was utilized to characterize the functional groups of the monomers TAPT and DHTA, TAPT-DHTA-COF, magnetic TAPT-DHTA-COF, and carboxylated magnetic spheres. As shown in Figure 2a, the peaks of C=N at 1620 cm^−1^ appeared in both TAPT-DHTA-COF and magnetic TAPT-DHTA-COF. Concurrently, the disappearance of the C=O (1670 cm^−1^) from DHTA and the N-H (3320 cm^−1^) from TAPT confirmed the formation of imine bonds between the amino and aldehyde monomers. Furthermore, the appearance of a vibration peak at 576 cm^−1^ in magnetic TAPT-DHTA-COF, corresponding to the Fe-O vibration of carboxylated magnetic spheres, verified the successful incorporation of magnetic particles into the COF framework. XRD was employed to examine the phase composition and crystalline structure of magnetic TAPT-DHTA-COF. As presented in Figure 2b, characteristic diffraction peaks at 2.8°, 4.9°, 5.6°, and 7.5° were assigned to the (100), (110), (200), and (120) crystal planes of TAPT-DHTA-COF, respectively. Additionally, peaks at 30.1°, 35.5°, 43.4°, 53.4°, 57.1°, and 62.8° corresponded to the (220), (311), (400), (422), (511), and (440) crystal planes of carboxylated magnetic spheres, indicating the well-preserved crystalline nature of the composite material.

Nitrogen adsorption–desorption experiments were conducted to evaluate the specific surface area and pore characteristics of magnetic TAPT-DHTA-COF, with the results shown in Figure 2c and Figure S6. The average pore diameter and specific surface area of TAPT-DHTA-COF were determined to be 3.3 nm and 397.7 m^2^ g^−1^, respectively, while the corresponding values for magnetic TAPT-DHTA-COF were 3.5 nm and 449.1 m^2^ g^−1^. These results indicate that the synthesized magnetic TAPT-DHTA-COF possesses a well-developed porous structure and a high specific surface area. The magnetization hysteresis curves of magnetic TAPT-DHTA-COF and carboxylated Fe_3_O_4_ are presented in Figure 2d. The saturation magnetization values of magnetic TAPT-DHTA-COF and carboxylated Fe_3_O_4_ were determined to be 8.5 emu g^−1^ and 71.8 emu g^−1^, respectively. Neither magnetization curve exhibited magnetic hysteresis or remanence, confirming that both materials possess excellent superparamagnetic properties, facilitating rapid magnetic separation.

The elemental composition of magnetic TAPT-DHTA-COF was examined by XPS, revealing characteristic peaks of C 1s, Fe 2p, N 1s, and O 1s in Figure 2e. The XPS spectrum of C 1s is shown in Figure 2f, with peaks at 284.18 eV and 285.78 eV attributed to C=C and C-N, respectively. These results indicate the successful formation of the aromatic framework of the COF. The O 1s spectrum further confirms that the oxygen species peaks at 531.48 eV and 532.98 eV correspond to C-O and C=O, respectively, which may originate from linking units within the framework (Figure 2i). In the N 1s spectrum, signals at 398.28 eV are assigned to C=N, directly confirming the formation of imine linkages (C=N), which are characteristic of imine-based COFs (Figure 2h). The Fe 2p spectrum reveals the coexistence of Fe(II) and Fe(III), indicating the successful incorporation of Fe_3_O_4_ nanoparticles, which impart magnetic properties to the COF (Figure 2g). The iron species are likely present as Fe-O bonds. The aforementioned characterization results proved that magnetic TAPT-DHTA-COF possessed a good crystal structure, a large specific surface area, and excellent magnetism, which provided a good material basis for the application of MSPE.

### 2.3. MSPE Conditions

Key experimental parameters were systematically optimized, including the adsorbent dosage, extraction time, sample solution pH, and ionic strength, as well as the type and volume of the elution solvent. In these single-factor optimization experiments, each condition was tested with three replicates, and the recovery rates under different experimental setups were evaluated.

**Figure 3:**
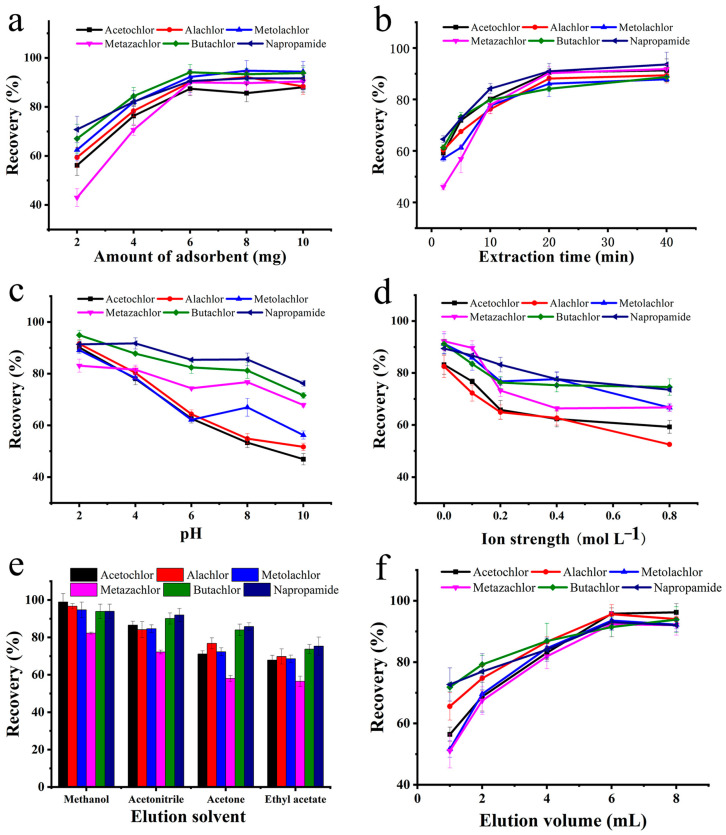
MSPE parameter optimization: (**a**) amount of adsorbent, (**b**) extraction time, (**c**) sample pH, (**d**) sample ion strength, (**e**) elution solvent, and (**f**) elution volume.

The influence of adsorbent dosage (2, 4, 6, 8, and 10 mg) on the recovery of six AHs was investigated. As shown in Figure 3a, the recoveries of all six herbicides were below 70% at an adsorbent dosage of 2 mg. Recoveries increased with higher adsorbent amounts but plateaued when the dosage was raised from 6 mg to 10 mg. This indicates that an insufficient quantity of adsorbent cannot provide adequate active sites, whereas 6 mg is sufficient for effective adsorption under the given sample concentration. To conserve materials, 6 mg was selected as the optimal dosage.

The extraction time was optimized over a range of 2–40 min. As depicted in Figure 3b, recoveries increased significantly as the extraction time was extended from 2 to 10 min. A further increase to 20 min resulted in continued but slower improvement. Beyond 20 min, no notable enhancement was observed. These results demonstrate the rapid adsorption kinetics of magnetic TAPT-DHTA-COF for the target herbicides, reflecting its strong affinity and enabling a shortened extraction time. Accordingly, 20 min was chosen as the optimal adsorption duration.

The impact of the sample pH (2.0–10.0) on the extraction efficiency is illustrated in Figure 3c. Recoveries decreased progressively with the increasing pH. This trend is likely attributable to the ionization behavior of AHs, whose pKa values range from −0.82 to 1.54. Under acidic conditions, the neutral molecular forms predominate, favoring adsorption onto the magnetic COF. In alkaline media, the ionization of herbicides reduces their affinity for the adsorbent, resulting in lower recoveries. Therefore, a sample pH of 2.0 was selected for subsequent experiments.

Sample solutions with NaCl concentrations of 0, 0.1, 0.2, 0.4, and 0.8 mol L^−1^ were prepared to evaluate the influence of ionic strength. As shown in Figure 3d, recoveries declined with the increasing salt concentration, indicating that higher ionic strength inhibits the adsorption of AHs onto magnetic TAPT-DHTA-COF. This may be explained by the hydrophilic nature of both the hydroxyl-rich adsorbent and the amide-containing analytes, wherein an elevated ionic strength could interfere with their interaction. Consequently, no salt addition was employed in further experiments.

The elution efficiency of four solvents (methanol, acetonitrile, acetone, and ethyl acetate) was compared (Figure 3e). Methanol demonstrated the highest elution capacity, followed by acetonitrile and acetone, while ethyl acetate performed the poorest. Thus, methanol was chosen as the eluent. Further investigation was conducted into its optimal volume over a range of 1, 2, 4, 6, and 8 mL. As presented in Figure 3f, recoveries increased progressively with the eluent volume up to 6 mL, beyond which no significant improvement was observed. Therefore, 6 mL of methanol was selected for elution.

Based on the above optimizations, the experimental conditions were established as an adsorbent dosage of 6 mg, an adsorption time of 20 min, a sample pH of 2.0, no ionic strength adjustment, and elution with 6 mL of methanol.

### 2.4. Method Validation Analysis

Matrix-matched calibration standards were prepared using green tea extract and analyzed by MSPE-GC-MS/MS. The linear range, R^2^, LOD, LOQ, and intra-day and inter-day precision for the six AHs are summarized in Table 1. All compounds showed excellent linearity in the range of 1–500 ng g^−1^, with an R^2^ between 0.9910 and 0.9982. The LODs ranged from 0.25 to 0.73 ng g^−1^, and the LOQs ranged from 0.83 to 2.32 ng g^−1^. Intra-day and inter-day RSDs were 2.3–5.7% and 1.3–6.1%, respectively.

The developed MSPE-GC-MS/MS method was applied to perform spiked recovery experiments using blank green tea and black tea samples. As summarized in Table 2, the recoveries of the six AHs in green tea ranged from 82.0% to 94.8%, with an RSD below 5.1%. In black tea samples, recoveries were between 80.1% and 92.2%, with RSDs less than 6.2%. The recovery results meet the performance criteria for pesticide residue analysis, as stipulated in the EU SANTE/11945/2015 guidelines.

### 2.5. Application of the MSPE-GC-MS/MS Method

The established approach was applied to screen the actual content of 10 kinds of green tea and black tea samples from the local market. As shown in Table S2, the six amide herbicides were detected in several tea samples, with concentrations ranging from 0.33 to 0.88 ng/g. The RSD values ranged from 2.9% to 8.3%, demonstrating an acceptable precision even at low concentration levels. Butachlor was the most frequently detected herbicide, found in 50% of the samples. The overall detection rate of herbicides was significantly higher in green teas than in black teas. This discrepancy may be attributed to differences in cultivation and management practices between the two tea types. As a non-fermented tea, green tea places higher demands on the appearance and safety of fresh leaves, which may lead to more frequent herbicide applications in its production areas to control weeds. In contrast, black tea-producing regions may employ different farming systems, or the fermentation process involved in black tea-processing could facilitate the transformation of certain herbicide residues, resulting in a relatively lower detection rate. All detected values were below 1 ng g^−1^, indicating that the residual levels were generally low. This demonstrates that the established analytical method has been successfully applied to the detection of actual tea samples, effectively quantifying multiple herbicide residues in various categories of tea. The method exhibits excellent applicability and reliability in practical samples, confirming its practical value as a tool for tea safety testing. Figure S7 displays the total ion chromatogram of the six AHs in the green tea matrix. The chromatographic peaks are sharp and symmetrical, indicating an excellent peak shape. Under the applied GC-MS conditions, all six compounds were well resolved.

### 2.6. Stability of Magnetic TAPT-DHTA-COF Adsorbent

Magnetic TAPT-DHTA-COF was dispersed separately in a 0.01 mol L^−1^ NaOH solution, 0.01 mol L^−1^ HCl solution, and different organic reagents (methanol and ethyl acetate) and maintained at room temperature for three days. Following magnetic separation, elution, and drying, the treated materials were analyzed by FTIR spectroscopy. As shown in Figure S8, the FTIR spectra of magnetic TAPT-DHTA-COF exposed to various conditions retained the characteristic absorption peaks observed in the untreated material, indicating that the synthesized composite possesses excellent chemical stability. This provides a robust material foundation for its application in MSPE sample pretreatment.

The prepared magnetic TAPT-DHTA-COF was employed as an adsorbent in MSPE and could be regenerated through cyclic adsorption–desorption operations. To assess its economic feasibility, reuse experiments were conducted using the same batch of materials, with recovery rates serving as the performance indicator. The sample solution consisted of green tea extract spiked with AHs at a concentration of 100 ng g^−1^. For each cycle, 6 mg of the adsorbent was used, and all experiments were performed in triplicate. After each MSPE cycle, the adsorbent was regenerated by sequential ultrasonication with 3 mL of methanol and 3 mL of ultrapure water for 5 min each before reuse in the subsequent extraction cycle. The variation in recovery rates of the six AHs as a function of reuse cycles is presented in Figure S9. As shown, the recoveries remained within the range of 80–100% during the first 15 cycles. However, upon further reuse, the recovery rates of alachlor, acetochlor, and metolachlor decreased to below 80%. Given that the sample matrix consisted of green tea extract, this decline in performance may be attributed to the competitive occupation of active adsorption sites by non-target matrix components. Consequently, the synthesized magnetic TAPT-DHTA-COF can be effectively reused for up to 15 cycles under the proposed method.

### 2.7. Adsorption Interaction Mechanism

Table S3 summarizes the physicochemical properties of the six target AHs, three glucocorticoids (prednisolone, triamcinolone, and hydrocortisone), and three polycyclic aromatic hydrocarbons (PAHs: phenanthrene, pyrene, and benzo[a]pyrene). As listed, the six AHs exhibit Log *K*_ow_ values ranging from 2.13 to 4.50, possess zero hydrogen-bond donors, and contain 3–4 hydrogen-bond acceptors. The three glucocorticoids have Log *K*_ow_ values between 1.16 and 1.62, with 3–4 hydrogen-bond donors and 5–6 hydrogen-bond acceptors. In contrast, the three PAHs show higher Log *K*_ow_ values (4.46–5.99) and lack both hydrogen-bond donors and acceptors.

As summarized in Table 3, the recoveries of the six AHs extracted by magnetic TAPT-DHTA-COF via MSPE ranged from 85.4% to 95.9%. The material also demonstrated a high extraction efficiency for the three glucocorticoids, with recoveries between 88.5% and 92.8%. In contrast, the recoveries for the three PAHs were comparatively low at 9.6–15.4%. Based on these results, the adsorption mechanism of magnetic TAPT-DHTA-COF toward AHs is proposed to involve the following processes. The carboxylated magnetic spheres possess inherent hydrophilicity. When incorporated into the magnetic TAPT-DHTA-COF, they enhance the overall hydrophilicity of the composite, promoting its uniform dispersion in aqueous media. Furthermore, magnetic TAPT-DHTA-COF is rich in hydroxyl groups, which readily engage in hydrogen-bonding interactions in aqueous environments. Both AHs and glucocorticoids contain hydrogen-bond acceptors in their molecular structures, enabling the formation of hydrogen bonds with the adsorbent. In contrast, PAHs lack both hydrogen-bond donors and acceptors, precluding such intermolecular hydrogen-bonding interactions. Therefore, hydrogen bonding is identified as the primary driving force for the adsorption process.

### 2.8. Comparison with Other Methods

The superiority of the established MSPE-GC-MS/MS method was further demonstrated by comparing it with other reported methods (Table S4). Firstly, in terms of pretreatment time, this work requires only 20 min, which is significantly shorter than many other methods, resulting in higher efficiency. Secondly, regarding the adsorbent dosage, this work uses merely 6 mg of magnetic TAPT-DHTA-COF adsorbent, which is substantially lower than the amounts used in other methods, indicating superior adsorption efficiency and cost-effectiveness. Additionally, this method achieves low LOD and LOQ, which are comparable to or better than several other techniques, while maintaining good recovery rates and acceptable precision. Furthermore, the method employs a minimal volume of organic reagent, reducing the solvent consumption and aligning with green analytical chemistry principles. Overall, this work presents a fast, sensitive, economical, and environmentally friendly approach suitable for the determination of target analytes in complex matrices.

## 3. Materials and Methods

### 3.1. Reagents and Instruments

Reagents and instruments are listed in the Supplementary Materials.

### 3.2. GC-MS/MS Analytical Conditions

A 100 mg L^−1^ mixed standard of six AHs (acetochlor, alachlor, metolachlor, metazachlor, butachlor, and napropamide) was prepared. A series of working standard solutions at concentrations of 1, 5, 10, 50, 100, 250, and 500 μg L^−1^ were obtained through serial dilution with ethyl acetate. All standard solutions were stored at 4 °C until use.

The analysis of the six AHs was conducted using an Agilent 7890B GC system (Agilent Technologies, Santa Clara, CA, USA) coupled with a 7000D triple quadrupole MS, equipped with an HP-5 capillary column (30 m × 0.25 mm, 0.25 µm film thickness). The GC temperature program comprised an initial isothermal stage at 100 °C for 1 min, followed by a linear increase to 180 °C at 25 °C min^−1^, a subsequent ramp to 210 °C at 3 °C min^−1^, and a final ascent to 280 °C at 20 °C min^−1^, with a concluding 5 min isothermal hold at this ultimate temperature. The mass spectrometric detection was conducted with ionization in the electron impact (EI) mode at 70 eV; the ion source temperature held at 230 °C, with high-purity helium as the carrier gas at a constant flow of 1.0 mL min^−1^ and argon as the collision gas. The mass spectrometric detection was performed in selected ion monitoring (SIM) mode. The detailed MS parameters are listed in Table S1.

### 3.3. Synthesis of Carboxylated Magnetic Spheres

Carboxylated magnetic spheres were prepared via two synthetic routes. In the first method, FeCl_3_·6H_2_O (270.3 mg, 1 mmol), Na_3_C_6_H_5_O_7_·2H_2_O (882.3 mg, 3 mmol), and urea (360.4 mg, 6 mmol) were accurately weighed into a 100 mL round-bottom flask. Ultrapure water (40 mL) was added, and the mixture was stirred magnetically at 1000 rpm for 2 min to obtain a clear, bright yellow solution. Subsequently, polyacrylamide (300 mg) was introduced, and stirring was continued at 1000 rpm for 55 min. The resulting mixture was then transferred to a 100 mL polytetrafluoroethylene (PTFE)-lined autoclave and reacted at 200 °C for 12 h. The formed magnetic spheres were isolated by magnetic separation, sequentially washed three times with ultrapure water and methanol, and finally dried under vacuum at 70 °C for 12 h.

For the alternative synthesis, FeCl_3_·6H_2_O (2.025 g), CH_3_COONH_4_ (5.781 g), and Na_3_C_6_H_5_O_7_·2H_2_O (0.6 g) were combined in a 250 mL round-bottom flask. Ethylene glycol (110 mL) was added, and the mixture was stirred at 1000 rpm for 1 h at room temperature. The homogeneous mixture was divided equally into two 100 mL PTFE-lined autoclaves and reacted at 200 °C for 16 h. The isolation, washing, and drying procedures were identical to those described for the first method.

### 3.4. Synthesis of Magnetic TAPT-DHTA-COF

The magnetic TAPT-DHTA-COF composite was fabricated via an in situ synthesis and assembly approach at room temperature, which involved amidation followed by a Schiff base reaction.

Magnetic spheres (50 mg) were first dispersed in dimethyl sulfoxide (DMSO) (25 mL) via ultrasonication for 30 min. Subsequently, Dicyclohexylcarbodiimide (206 mg) and N-hydroxysuccinimide (288 mg) were introduced into the dispersion [31,32]. The mixture was agitated on a tumbler shaker operating at 40 rpm for 30 min. Following the addition of TAPT (180 mg, delivered as a solution in 5 mL of DMSO), the reaction mixture was maintained under the prevailing conditions with subsequent continuation of the reaction for 24 h. The resultant product was isolated via magnetic separation and subsequently subjected to three sequential washing cycles using DMSO, ultrapure water, and ethanol, with two repetitions for each solvent.

The product obtained from the amidation step (20 mg) was redispersed in a mixed solvent consisting of dichlorobenzene (10 mL) and n-butanol (10 mL) by ultrasonication for 30 min. This dispersion was transferred to a vertical rotary mixer and stirred at 60 rpm. DHTA (50 mg) was introduced, and the reaction was carried out for 10 min, after which 6 mol L^−1^ HAc (2 mL) was added and allowed to react for 1 h. Finally, TAPT (70 mg) was incorporated, and the reaction was continued for 24 h. The batch-to-batch reproducibility of the MCOF synthesis was evaluated using three independently synthesized batches. The results are provided in Figure S1. For comparative analysis, two control samples were prepared, one being a magnetic COF synthesized via direct Schiff base reaction between magnetic spheres TAPT and DHTA without the preceding amidation step, and the other being a non-magnetic TAPT-DHTA-COF synthesized via Schiff base reaction between TAPT and DHTA in the absence of magnetic spheres. All final products were collected, subjected to three washing cycles with methanol, and dried in a vacuum oven at 70 °C for 12 h.

### 3.5. Optimization of MSPE Parameters

Key parameters affecting the efficiency of MSPE were systematically optimized. These included adsorbent dosage (ranging from 2 to 10 mg), extraction time (2–40 min), sample pH (2.0–10.0), ionic strength (adjusted using 0–0.8 mol L^−1^ NaCl), type of elution solvent (methanol, acetonitrile, acetone, or ethyl acetate), and elution volume (1–8 mL). For these optimization experiments, water samples were spiked with AHs at a concentration of 100 ng L^−1^. Each condition was tested in triplicate, and recovery rates were evaluated as the primary performance metric.

### 3.6. MSPE-GC-MS/MS Procedure

Magnetic TAPT-DHTA-COF (6 mg) was introduced into a glass bottle containing 40 mL of the sample solution. The bottle was placed on a vertical rotary mixer and agitated at 80 rpm to facilitate adsorption. Following the adsorption step, the magnetic adsorbent was isolated using an external magnetic field. An elution solvent (methanol, 6 mL) was then added to the separated adsorbent, and the mixture was subjected to ultrasonic-assisted elution for 5 min. The eluent was collected via magnetic separation, transferred to a 10 mL polyethylene tube, and dried under a gentle nitrogen stream at 40 °C. The resulting residue was reconstituted in ethyl acetate (0.5 mL) by ultrasonication for 5 min. The final solution was filtered through a 0.22 μm microporous membrane prior to GC-MS/MS analysis. For potential reuse, the recovered magnetic adsorbent was washed sequentially with methanol (3 mL) and ultrapure water (3 mL).

### 3.7. Real Sample Preparation

Tea samples were ground into a homogeneous powder. A precisely weighed portion (0.50 g) of the tea powder was transferred into a 50 mL centrifuge tube, followed by the addition of acidified acetonitrile (1% formic acid, 2.5 mL). The mixture was subjected to vortex oscillation for 5 min and subsequently centrifuged at 8000 *g* rpm for 5 min. This extraction process was repeated once. The combined supernatants were diluted to a final volume of 40 mL with ultrapure water, and the pH was adjusted to 2.0.

### 3.8. Method Validation

To establish the calibration curve of the method, a series of matrix-matched standards were prepared by spiking blank green tea samples with the six target AHs at concentrations of 1, 5, 10, 50, 100, 250, and 500 ng g^−1^. The tea sample was subjected to a series of steps including adsorption, magnetic separation, elution, and reconstitution prior to analysis by GC-MS/MS. These curves were employed to determine the linear dynamic range, correlation coefficient (R^2^), limit of detection (LOD), and limit of quantification (LOQ). To assess accuracy and precision, recovery experiments were conducted by spiking blank green tea and black tea samples at three concentration levels (5, 50, and 250 ng g^−1^), with three replicate analyses per level. Recovery rates and the corresponding relative standard deviations (RSDs) were calculated accordingly.

### 3.9. Adsorption Experiment

Analysis of the physicochemical properties of AHs reveals that these compounds exhibit a certain degree of hydrophilic nature, possessing an amide functional group in their chemical structure while also containing multiple hydrogen-bond acceptors. Therefore, to investigate the interaction forces between magnetic TAPT-DHTA-COF and AHs, three hydroxyl-containing glucocorticoids (prednisolone, triamcinolone, and hydrocortisone) and three non-hydroxylated polycyclic aromatic hydrocarbons (phenanthrene, pyrene, and benzo[a]pyrene) were introduced as comparative compounds. Using magnetic TAPT-DHTA-COF as the adsorbent, spiked recovery experiments were performed on these two groups of compounds to elucidate the potential adsorption mechanism between TAPT-DHTA-COF and AHs.

## 4. Conclusions

In summary, starting from the physicochemical properties of such polar compounds of AHs, we designed and synthesized a novel magnetic TAPT-DHTA-COF material with certain hydrophilic characteristics. This material was prepared at room temperature by in situ loading of hydroxyl-rich TAPT-DHTA-COF onto carboxyl-functionalized magnetic spheres, which can stably disperse in aqueous media, followed by the optimization of their binding mode. Due to the strong hydrogen-bonding interactions between the magnetic TAPT-DHTA-COF and AHs, the newly established MSPE method requires only 6 mg of adsorbent, can be reused for up to 15 cycles, and offers rapid and simple operation, demonstrating favorable economic applicability. The established MSPE-GC-MS/MS method can be extended beyond tea samples and shows potential for highly sensitive determination of AHs in other food or environmental matrices in the future. This study provides insights and technical support for the development and application of novel magnetic extraction materials.

## Figures and Tables

**Table 1:** Analytical data for the proposed method.

Compound	Linear Range (ng g^−1^)	R^2^	LOD (ng g^−1^)	LOQ (ng g^−1^)	RSD (%, *n* = 3)	
					Intra-Day	Inter-Day
Acetochlor	1–500	0.9982	0.58	1.96	2.3	1.3
Alachlor	1–500	0.9962	0.35	1.18	5.7	3.9
Metolachlor	1–500	0.9961	0.25	0.83	3.9	4.3
Metazachlor	1–500	0.9910	0.50	1.67	4.1	6.1
Butachlor	1–500	0.9925	0.73	2.32	2.8	3.1
Napropamide	1–500	0.9933	0.53	1.75	5.2	4.5

**Table 2:** Recovery experiment of tea samples (*n* = 3).

Compound	Spiked Concentration (ng g^−1^)	Recovery ± RSD (%)	
		Green Tea	Black Tea
Acetochlor	5	87.4 ± 2.8	80.1 ± 2.3
	50	85.6 ± 3.5	90.4 ± 1.5
	250	88.0 ± 2.1	91.2 ± 4.4
Alachlor	5	88.4 ± 3.3	82.5 ± 4.3
	50	90.1 ± 3.7	88.1 ± 0.9
	250	92.1 ± 2.5	89.4 ± 3.0
Metolachlor	5	82.0 ± 1.6	87.8 ± 1.9
	50	92.2 ± 2.8	85.9 ± 4.8
	250	94.8 ± 4.1	91.2 ± 3.8
Metazachlor	5	90.0 ± 5.1	83.1 ± 2.5
	50	89.7 ± 3.1	92.2 ± 3.6
	250	90.3 ± 2.9	89.6 ± 2.7
Butachlor	5	94.1 ± 3.2	83.5 ± 3.8
	50	93.3 ± 2.3	87.8 ± 6.2
	250	93.8 ± 2.4	91.2 ± 1.8
Napropamide	5	82.1 ± 4.5	83.2 ± 2.7
	50	90.5 ± 4.7	86.7 ± 4.5
	250	91.6 ± 2.5	89.4 ± 1.9

**Table 3:** The recoveries of different compounds by magnetic TAPT-DHTA-COF-based MSPE.

Compound	Structure	Recovery ± RSD (%)	Compound	Structure	Recovery ± RSD (%)
Acetochlor	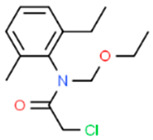	88.9 ± 2.6	Prednisolone	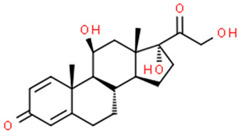	91.6 ± 2.8
Alachlor	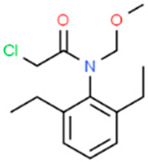	90.1 ± 1.9	Triamcinolone	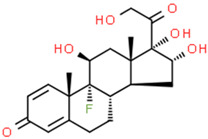	92.8 ± 4.4
Metolachlor	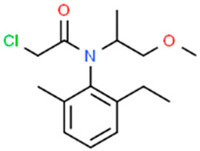	85.4 ± 5.0	Hydrocortisone	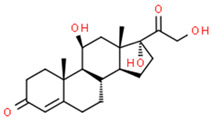	88.5 ± 2.4
Metazachlor	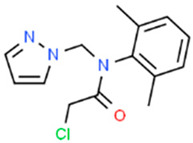	93.4 ± 2.9	Phenanthrene	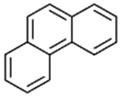	15.4 ± 5.0
Butachlor	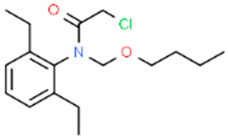	92.2 ± 2.1	Pyrene	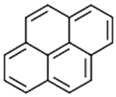	11.6 ± 2.3
Napropamide	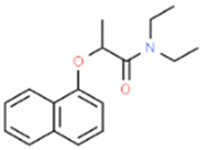	95.9 ± 3.9	Benzo[a]pyrene	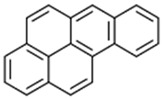	9.6 ± 3.1

## Data Availability

Data will be made available on request.

## Supplementary Materials

The following supporting information can be downloaded at: https://www.mdpi.com/article/10.3390/molecules31111940/s1, Figure S1: Batch-to-batch reproducibility of the MCOF material; Figure S2: SEM image of magnetic sphere synthesized in the organic phase (ethylene glycol); Figure S3: Comparison of time-dependent dispersion of two magnetic spheres in water; Figure S4: SEM image of magnetic TAPT-DHTA-COF 2; Figure S5: EDS elemental overlay mapping of magnetic TAPT-DHTA-COF 1; Figure S6: Nitrogen adsorption-desorption isotherms with pore size distributions; Figure S7: Total ion flow chromatogram of six AHs:1 acetochlor, 2 alachlor, 3 metolachlor, 4 metazachlor, 5 butachlor, 6 napropamide; Figure S8: FTIR spectra analysis of magnetic TAPT-DHTA-COF after treatment under various conditions; Figure S9: Relationship between the recovery of six AHs and the number of MSPE cycles; Table S1: Retention time and SIM parameters of six Ahs; Table S2: Contents of six AHs in real tea samples; Table S3: Physicochemical data of different compounds; Table S4: Method comparison for the determination of six AHs. References [33,34,35,36,37,38,39] are cited in the Supplementary Materials.
