# Supplementary material for: Novel Magnetic Covalent Organic Frameworks Fabricated Through In Situ Synthesis and Assembly for the Efficient Extraction and Enrichment of Six Amide Herbicides
Source: Molecules. 2026 Jun 3;31(11):1940. doi: 10.3390/molecules31111940 (PMC13257943; doi:10.3390/molecules31111940)
Supplement: Supplementary file 1 [file molecules-31-01940-s001.zip › molecules-4268056-supplementary.pdf]

Novel Magnetic Covalent Organic Frameworks Fabricated Through In Situ Synthesis and Assembly for the Efficient Extraction and Enrichment of Six Amide Herbicides

n

1. The chemicals and instruments

The COF monomers of 2,4,6-tris(4-aminophenyl)-1,3,5-triazine (TAPT, 98.0%), sodium citrate dihydrate ($Na_3C_6H_5O_7·2H_2O$, 99%), urea (99%), o-Dichlorobenzene and n-Butanol were purchased from Xiensi Biochemical Technology Co., Ltd. (Tianjin,China). The COF monomers of 2,5-dihydroxyterephthalaldehyde (DHTA, 98%) was supplied by Alfa Chemical Co., Ltd. (Zhengzhou, China). Dicyclohexylcarbodiimide (DCC, 98%), N-hydroxysuccinimide (NHS, 99%), ammonium acetate (CH_3COONH_4, ⩾99%) and dimethyl sulfoxide (DMSO, ⩾99.5%) were provided by Aladdin Biochemical Technology Co., Ltd. (Shanghai, China). Iron (III) chloride hexahydrate ($FeCl_3·6H_2O$, ⩾99%), Acetic acid (HAc,⩾99.8%) and anhydrous ethanol (⩾95%) were acquired from Sinopharm Chemical Reagent Co., Ltd. (Shanghai, China). Polyacrylamide was obtained from Acros Organics. Ethylene glycol and ethyl acetate were purchased from Tianjin Chemical Reagent Research Institute Co., Ltd. (Tianjin, China). Methanol (⩾99.9%) and acetonitrile (⩾99.9%) were procured from Merck Millipore (Merck KGaA, Germany). Acetochlor, alachlor, metolachlor (⩾97%), metazachlor, butachlor (⩾95%), napropamide, Prednisolone (⩾ 98%), triamcinolone (⩾99%), hydrocortisone (⩾98%), phenanthrene (⩾97%),

pyrene (⩾ 99%) and benzo[a]pyrene were supplied by Aladdin Biochemical Technology Co., Ltd. (Shanghai, China). Ultrapure water was supplied by the Milli-Q (Millipore, USA).

Scanning electron microscopy (SEM) images were studied using a SUPRA 55 instrument (Zeiss, Oberkochen, Germany). Transmission electron microscopy (TEM) images were recorded using the HT-7700 (Hitachi New Technology, Tokyo, Japan). Fourier transform infrared (FTIR) in the wavelength range of 500-4000 cm^{-1} were collected using a Nicolet 710 spectrometer (Thermo Scientific, Waltham, MA, USA). The X-ray diffraction (XRD) data was analyzed using a D/MAX-r8 diffractometer (Rigaku, Tokyo, Japan). X-ray photoelectron spectroscopy (XPS) pattern was determined using a EACALAB Xi+ system (Thermo Scientific, Waltham, MA, USA). The N_2 adsorption-desorption isotherm was measured by the ASAP 2460 analyzer (Micromeritics, Norcross, GA, USA). Magnetization curves were characterized using a SQUID-VSM magnetometer (Quantum Design, San Diego, CA, USA).

2. Supplementary figures and tables

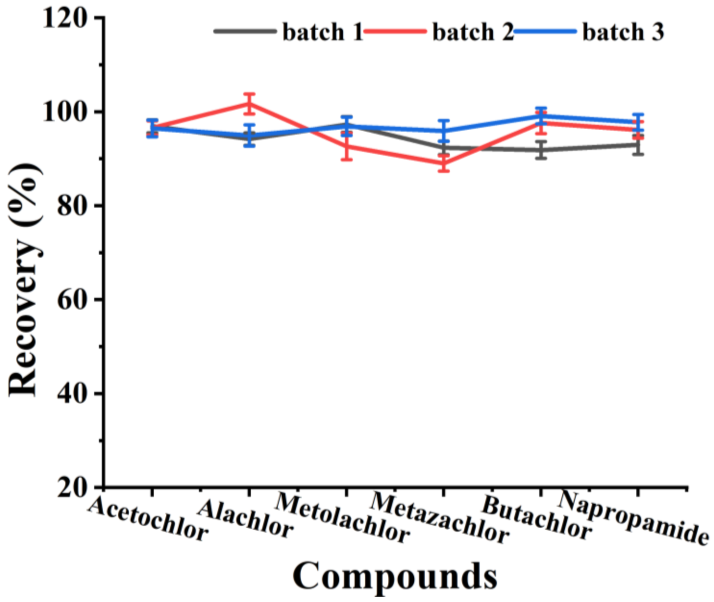

Figure S1 Batch-to-batch reproducibility of the MCOF material.

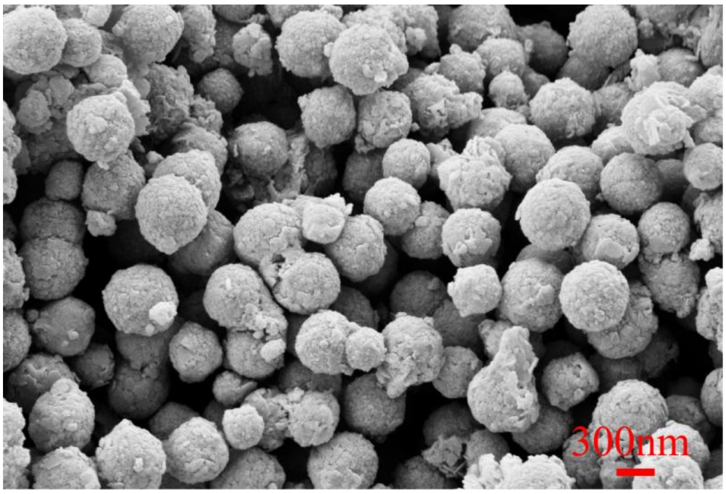

Figure S2 SEM image of magnetic sphere synthesized in the organic phase (ethylene glycol).

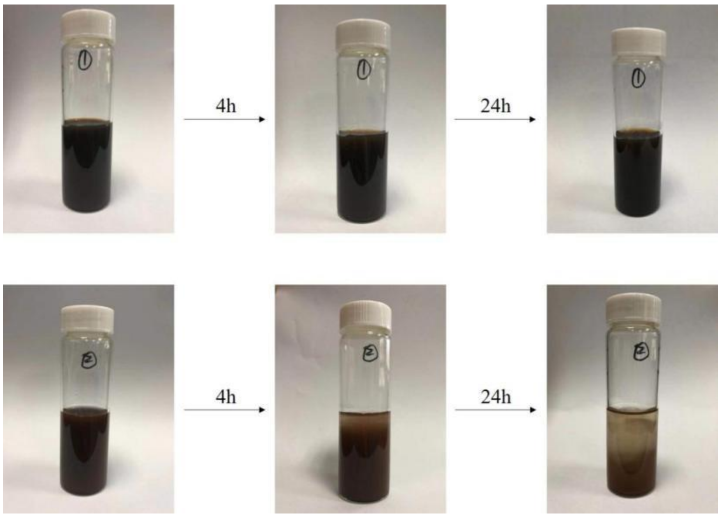

Figure S3 Comparison of time-dependent dispersion of two magnetic spheres in water.

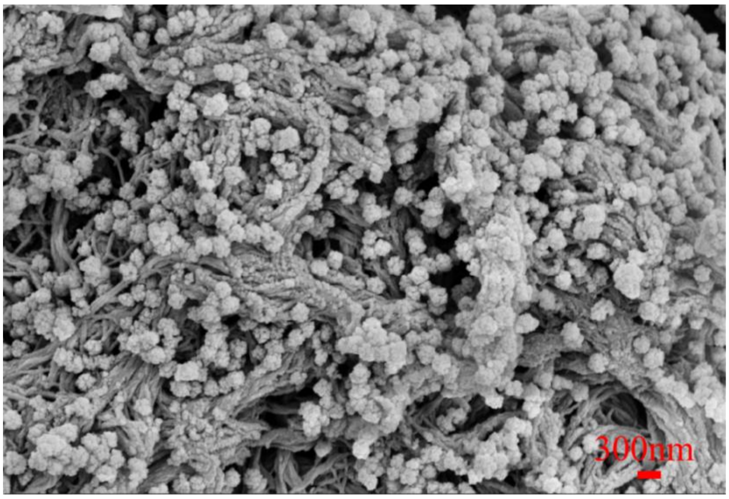

Figure S4 SEM image of magnetic TAPT-DHTA-COF 2.

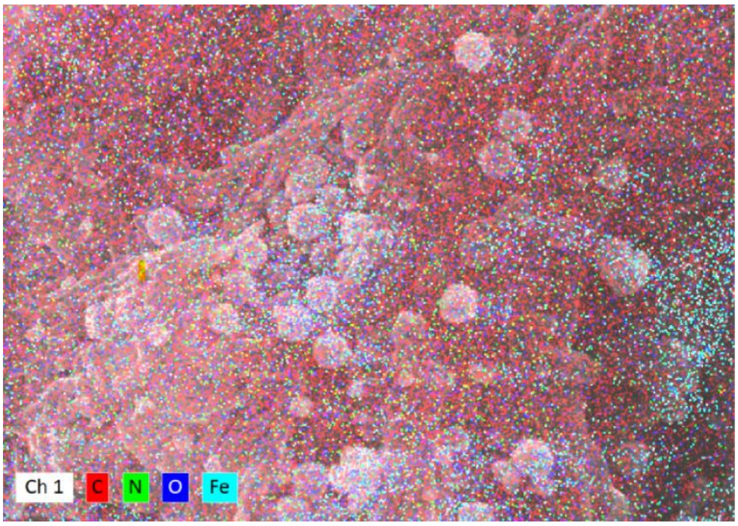

Figure S5 EDS elemental overlay mapping of magnetic TAPT-DHTA-COF 1

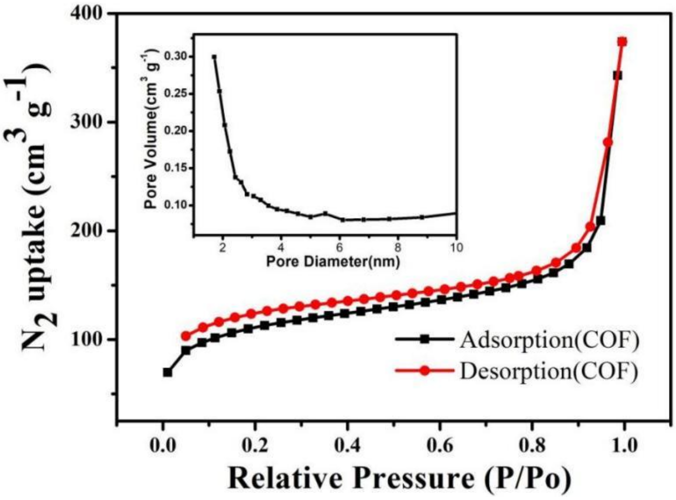

Figure S6 Nitrogen adsorption-desorption isotherms with pore size distributions.

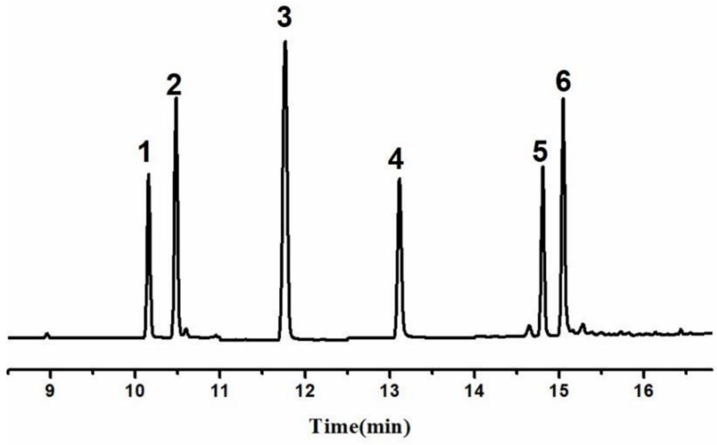

Figure S7 Total ion flow chromatogram of six AHs:1 acetochlor, 2 alachlor, 3 metolachlor, 4 metazachlor, 5 butachlor, 6 napropamide.

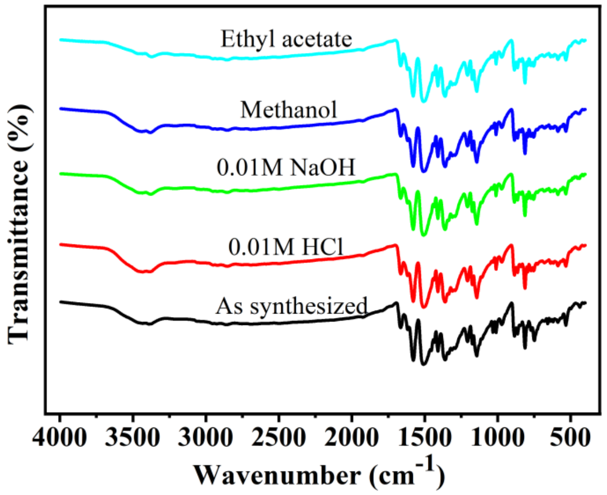

Figure S8 FTIR spectra analysis of magnetic TAPT-DHTA-COF after treatment under various conditions.

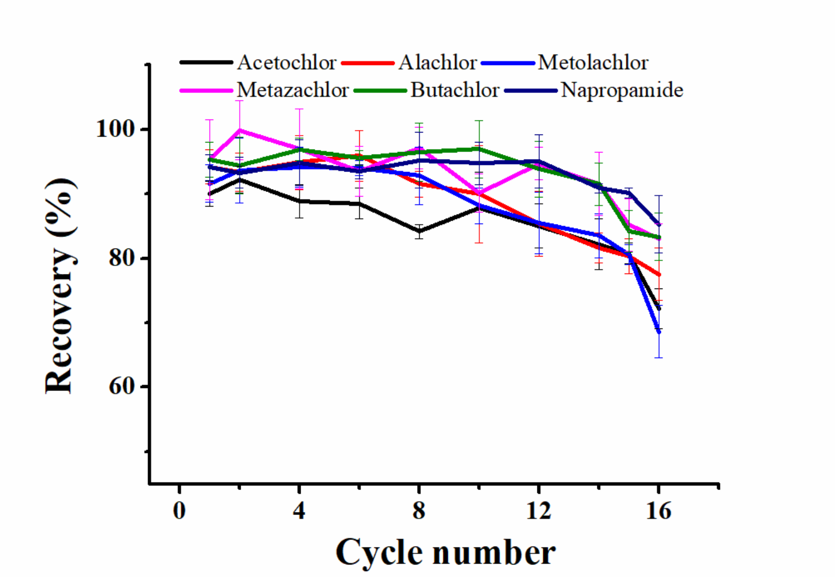

Figure S9 Relationship between the recovery of six AHs and the number of MSPE cycles.

Table S1. Retention time and SIM parameters of six AHs.

Compounds	Retention time (min)	Qualitative ion (m/z)	Quantitative ion (m/z)	CE (v)
Acetochlor	10.15	146.2	162.1	70
Alachlor	10.47	160.2	188.2	70
Metolachlor	11.76	238.2	162.2	70
Metazachlor	13.11	209.2	133.2	70
Butachlor	14.80	160.2	176.2	70
Napropamide	15.04	72.2	128.2	70

Table S2. Contents of six AHs in real tea samples.

Type		The content of AHs /ng g^{-1}± RSD (%, *n*=3)					
		Acetochlor	Alachlor	Metolachlor	Metazachlor	Butachlor	Napropamide
Gree-n tea	Sample 1	0.72 ± 5.6	n.d.	0.33 ± 7.9	n.d.	0.88 ± 3.1	n.d.
	Sample 2	0.65 ± 4.9	n.d.	n.d.	n.d.	n.d.	0.62 ± 4.3
	Sample 3	n.d.	n.d.	0.45 ± 6.6	n.d.	n.d.	n.d.
	Sample 4	n.d.	n.d.	n.d.	n.d.	0.79 ± 4.2	n.d.
	Sample 5	0.66 ± 3.0	n.d.	0.41 ± 8.3	n.d.	0.80 ± 3.3	n.d.

Type		The content of AHs /ng g^{-1}± RSD (%, n=3)					
		Acetochlor	Alachlor	Metolachlor	Metazachlor	Butachlor	Napropamide
Black tea	Sample 6	n.d.	n.d.	n.d.	n.d.	n.d.	n.d.
	Sample 7	n.d.	n.d.	n.d.	n.d.	n.d.	0.57 ± 7.4
	Sample 8	0.59 ± 4.1	n.d.	n.d.	n.d.	0.76 ± 2.9	n.d.
	Sample 9	n.d.	n.d.	n.d.	n.d.	0.78 ± 5.7	n.d.
	Sample 10	n.d.	n.d.	n.d.	n.d.	n.d.	n.d.

n.d.: not detected.

Table S3. Physicochemical data of different compounds.

Compounds	Molecular weight	H bond acceptors	H bond donors	Log *Kow*
Acetochlor	269.8	3	0	3.03
Alachlor	269.8	3	0	3.52
Metolachlor	283.8	3	0	3.13
Metazachlor	277.7	4	0	2.13
Butachlor	311.8	3	0	4.50
Napropamide	271.4	3	0	3.36
Prednisolone	360.4	5	3	1.62
Triamcinolone	394.4	6	4	1.16
Hydrocortisone	362.5	5	3	1.61
Phenanthrene	178.2	0	0	4.46
Pyrene	202.3	0	0	4.88
Benzo[a]pyrene	252.3	0	0	5.99

Table S4. Method comparison for the determination of six AHs.

Instrument	Pretreatment method	Consumption of organic reagent	Extraction time	Adsorbent type/dosage	LOD/LOQ	Recovery	Precision（RSD,%)	Reference
GC-MS	HS-SPME	20 mL milk / 8 mL acetonitrile	20 min	Urchin-like ZnO/NiO (50) microspheres /65 µm	LOD 0.003-0.025 ng L^{-1}, LOQ 0.01-0.1 ng L^{-1}	70.3%-114.1%	Intra-day: 2.4%-4.9%, inter-day: 2.5%-8.8%	[1]
GC-ECD	Ultrasound-assisted extraction + SPE	5 g fish meat / 20 mL n-hexane+ 3 mL dichloromethane/n-hexane (1:1, v/v)	40 min	SPE column packing/ 500 mg	LOD 0.19-0.42 µg kg^{-1}, LOQ 0.63-1.39 µg kg^{-1}	71.2%-92.6%	Intra-day:1.27%-4.36%, inter-day: 2.12%-4.74%	[2]
HPLC-FLD	SPE	5g sample / 20 mL acidulated acetonitrile	28 min	MI-COF/0.5mg	LOD 0.003-0.05 ng mL^{-1}, LOQ 0.01-0.16 ng mL^{-1}	80.4%-110.7%	Intra-day: 2.2%-2.9%, inter-day: 2.9%-3.5%	[3]
GC-MS/MS	MSPE	Acetonitrile/ethanol mixture (2:8, v/v)	15 min	Magnetic dummy molecularly imprinted polymers/50 mg	LOD 0.061 µM	85.59%-99.74%	< 8.57%	[4]
HPLC-MS/MS	MSPE	2.5 mL acetonitrile for desorption	18 min	Fe_3O_4@SiO_2-NH_2@Ph-POP/ 16 mg	LOD 0.015-1.412 µg L^{-1}, LOQ 0.049-4.707 µg L^{-1}	65.06-101.95%	Intra-day: < 9.89%, inter-day: < 10.54%	[5]
GC-MS	SPE	10 g field soil /20 mL methanol	30 min	MIL-101(Cr)/200 mg	LOD 0.25-0.45 µg kg^{-1}	86.3%-102.3%	Intra-day: 2.23%-4.38% inter-day: 2.07%-3.05%	[6]
GC-MS	SPE	1.0 g rice bran/15 mL chloroform-methanol (2:1, v/v)	1h	Strata silica column/500 mg	LOD 0.348-1.17 µg mL^{-1} LOQ 1.16-3.88 µg mL^{-1}	88.9%-104%	Intra-day : 0.94%-1.68%, inter-day: 1.50%-3.72%	[7]
GC-MS	MSPE	0.5 g tea / 5 mL acidified acetonitrile (1% formic acid, 2.5 mL)	20 min	Magnetic TAPT-DHTA-COF /6 mg	LOD 0.25-0.73 ng kg^{-1}, LOQ 0.83-2.32 ng g^{-1}	80.1%-94.8%	Intra-day:2.3%-5.7%, inter-day: 1.3%-6.1%	This work

References

1. Zhu, S.P.; Song, Z.; Wang, Y.M.; Zhu, J.W.; Hao, Y.G.; Lou, X.J.; Lu, M.H. Defective porous urchin-like ZnO/NiO microspheres-coated solid-phase microextraction fiber for analysis of trace polychlorinated biphenyls in milk. *J. Hazard. Mater.* **2024**, 480, 136362.
2. Qu, Z.P.; Bai, X.Z.; Zhang, T.; Yang, Z.G. Ultrasound-assisted extraction and solid-phase extraction for the simultaneous determination of five amide herbicides in fish samples by gas chromatography with electron capture detection. *J. Sep. Sci.* **2017**, 40, 1142–1149.
3. Su, L.H.; Qian, H.L.; Yang, C.; Wang, C.X.; Wang, Z.Y.; Yan, X.P. Surface imprinted-covalent organic frameworks for efficient solid-phase extraction of fluoroquinolones in food samples. *J. Hazard. Mater.* **2023**, 459, 132031.
4. Zeng, S.L.; Chen, Z.X.; Huang, L.; Li, C.H.; Wang, P.; Qin, D.L.; Gao, L. A highly efficient and selective rapid detection method applied to the detection of amide herbicides in fish serum. *Food Chem.* **2024**, 449, 139215.
5. Zhang, C.; Li, S.; Wu, J.; Ping, T.; Ma, L.; Wang, K.; Lian, K.Q. Developing a hydroxyl-functionalized magnetic porous organic polymer combined with HPLC-MS/MS for determining 31 amide herbicides in fruit wine. *Food Chem*. **2023**, 403, 134442.
6. Cai, Y.J.; Li, L.Y.; Zhang, J.; Li, Z.J.; Zhang, F.M.; Xu, Y.Q.; Tai, Z.G. Development of a MOF-based SPE method combined with GC-MS for simultaneous determination of alachlor, acetochlor and pretilachlor in field soil. *Environ. Monit. Assess*. **2023**, 195, 569.
7. Feng, S.M.; Wang, L.L.; Shao, P.; Lu, B.Y.; Chen, Y.F.; Sun, P.L. Simultaneous analysis of free phytosterols and phytosterol glycosides in rice bran by SPE/GC-MS. *Food Chem*. **2022**, 387, 132742.
